# Titanium micro-nano textured surface with strontium incorporation improves osseointegration: an *in vivo* and *in vitro* study*

**DOI:** 10.1590/1678-7757-2024-0144

**Published:** 2024-09-16

**Authors:** Pio Moerbeck da COSTA, Camila Chiérici MARCANTONIO, Diego Pedreira de OLIVEIRA, Maria Eduarda Scordamaia LOPES, Julio Cesar Sanchez PUETATE, Luan Viana FARIA, Letícia de Freitas CARVALHO, Rafael Scaf de MOLON, Idelmo Rangel GARCIA, Andressa Vilas Bôas NOGUEIRA, James DESCHNER, Joni Augusto CIRELLI

**Affiliations:** 1 Universidade Estadual Paulista Faculdade de Odontologia de Araraquara Araraquara São Paulo Brasil Universidade Estadual Paulista - UNESP, Faculdade de Odontologia de Araraquara, Departamento de Diagnóstico e Cirurgia, Araraquara, São Paulo, Brasil.; 2 Extremus Smart Surface São Carlos São Paulo Brasil Extremus Smart Surface, São Carlos, São Paulo, Brasil.; 3 Universidad San Francisco de Quito Escuela de Odontología Quito Pichincha Ecuador Universidad San Francisco de Quito USFQ, Escuela de Odontología, Departmento de Periodoncia, Quito, Pichincha, Ecuador.; 4 Universidade Estadual Paulista Faculdade de Odontologia de Araraçatuba Araçatuba São Paulo Brasil Universidade Estadual Paulista - UNESP, Faculdade de Odontologia de Araraçatuba, Departamento de Diagnóstico e Cirurgia, Araçatuba, São Paulo, Brasil.; 5 University Medical Mainz Department of Periodontology and Operative Dentistry Germany University Medical Mainz, Center of the Johannes Gutenberg University, Department of Periodontology and Operative Dentistry, Germany.

**Keywords:** Osseointegration, Titanium implant, Dental implant, Strontium, Surface modification, Animal model

## Abstract

**Objectives:**

This study aimed to investigate the osseointegration of titanium (Ti) implants with micro-nano textured surfaces functionalized with strontium additions (Sr) in a pre-clinical rat tibia model.

**Methodology:**

Ti commercially pure (cp-Ti) implants were installed bilaterally in the tibia of 64 Holtzman rats, divided into four experimental groups (n=16/group): (1) Machined surface - control (C); (2) Micro-nano textured surface treatment (MN); (3) Micro-nano textured surface with Sr2+ addition (MNSr); and (4) Micro-nano textured surface with a higher complementary addition of Sr2+ (MNSr+). In total, two experimental euthanasia periods were assessed at 15 and 45 days (n=8/period). The tibia was subjected to micro-computed tomography (μ-CT), histomorphometry with the EXAKT system, removal torque (TR) testing, and gene expression analysis by PCR-Array of 84 osteogenic markers. Gene expression and protein production of bone markers were performed in an in vitro model with MC3T3-E1 cells. The surface characteristics of the implants were evaluated by scanning electron microscopy (SEM), energy-dispersive spectroscopy (EDS), and laser scanning confocal microscopy.

**Results:**

SEM, confocal, and EDS analyses demonstrated the formation of uniform micro-nano textured surfaces in the MN group and Sr addition in the MNSr and MNSr+ groups. TR test indicated greater osseointegration in the 45-day period for treated surfaces. Histological analysis highlighted the benefits of the treatments, especially in cortical bone, in which an increase in bone-implant contact was found in groups MN (15 days) and MNSr (45 days) compared to the control group. Gene expression analysis of osteogenic activity markers showed modulation of various osteogenesis-related genes. According to the in vitro model, RT-qPCR and ELISA demonstrated that the treatments favored gene expression and production of osteoblastic differentiation markers.

**Conclusions:**

Micro-nano textured surface and Sr addition can effectively improve and accelerate implant osseointegration and is, therefore, an attractive approach to modifying titanium implant surfaces with significant potential in clinical practice.

## Introduction

Since the publication of Brånemark’s pioneering research on osseointegration, the field of implant dentistry has evolved into a well-established and highly predictable oral rehabilitation approach. However, with an increasingly aging population, several challenges persist, such as poor bone conditions that contribute to clinical failure.^1^ Over the past decades, experimental research has focused on surface modifications of dental implants and biofunctionalization, aiming to overcome these challenges.^2^ In this regard, the main changes are related to surface texture, wetting capacity, and chemical composition of the surfaces, which are known to be essential factors for successful bone integration. Moreover, it is well established that a moderately rough surface results in more bone formation and increased bone-to-implant contact compared to smooth surfaces. In this context, various approaches and surface modifications have been investigated to achieve rapid and durable bone integration.^2,3^

The surface characteristics of implants impact the extent of direct contact with bone, the rate of surrounding bone growth, and the quality of the mechanical bond strength between bone and implant.^4^ Research has evidenced that micro-nano surface structures remarkably enhance extracellular matrix synthesis by adherent cells. This leads to a more responsive and reliable integration with bone than conventional machined surfaces.^4,5^

Surface modification of titanium (Ti) endosseous implants at the micro-nano-scale level can potentially lead to changes not only in the topography, but also in the chemical composition of the surface. In this context, strontium (Sr), an alkaline-earth metal with atomic number 38, has shown promise for incorporation into dental implants.^6^ Similar to calcium (Ca), Sr can be incorporated into the mineral matrix of bone.^11^ Moreover, Sr can reduce bone resorption by inhibiting osteoclast activity and improve bone formation by stimulating osteoblast activity.^9^

The release of Sr in an appropriate dose directly at the tissue-implant interface can improve implant osseointegration, and several recent studies have evaluated the beneficial impacts of this delivery in biomaterials.^6^ Based on the hypothesis that the combination of a Sr-doped micro-nano Ti surface may enhance the material’s capacity of promoting osteogenesis, thus achieving rigid implant osseointegration, *in vivo* studies are required. Thus, our study aimed to evaluate the effects of micro-nano strontium-loaded surface implants, in an *in vivo* model. In addition, evaluation of the gene expression and protein production of some osteogenic markers were analyzed *in vitro* using MCT3-E1 pre-osteoblastic lineage cells.

## Methodology

### Implants, surface coating, and characterization

Implants with 2.2 mm in diameter and 4 mm in length were fabricated from commercially pure titanium bars (grade II) specifically for this study (Implalife Biotechnology, Araçatuba-SP, Brazil). After machining, implants received specific surface treatments (Extremus Smart Surface, São Carlos-SP, Brazil) according to the four experimental groups: Micro-nano textured surface (MN); Micro-nano textured surface with strontium addition (MNSr); Micro-nano textured surface with strontium plus (MNSr+), with higher amount of Sr than MNSr surfaces; and Control (C) group, without any additional treatment, maintaining the surface texture resulted from the machining process. After surface treatment, the samples were sterilized by gamma radiation (25 kGy, CETER/IPEN, São Paulo-SP, Brazil) and the surface characteristics of the implants were evaluated by scanning electron microscopy (SEM), energy-dispersive spectroscopy (EDS) analysis, and laser scanning confocal microscopy. cp-Ti discs 13 mm in diameter and 2 mm in thickness received the same treatments and were used for the *in vitro* experiments.

### In vivo experiments

#### Sample size calculation

For animal experiments, sample size was estimated using data from a similar previous study that used the same experimental model.^15^ The primary outcome selected was the Bone Implant Contact (BIC). With a power of 80% and an alpha level set at 0.05, a minimum of seven implants per group was necessary to compare the four different groups.

#### Animals and surgical procedures

In total, the study included 64 male Holtzman albino rats (*Rattus norvegicus*), aged 3 months, and with body weight ranging from 300 to 350 grams. The implants were installed bilaterally in the tibia. The animals were randomly distributed equally in four groups according to the surface treatments described above (by simple randomization), totalizing 16 animals per group, and 128 implants installed. A total of four animals were kept per cage under climate-controlled conditions (25 °C, 55% humidity, 12/12 h light-dark cycle). Free access to tap water and feed was maintained during all the study. All experiments followed the Animal Research: Reporting of In Vivo Experiments (ARRIVE) 2.0 guidelines and were approved by the institution’s Ethics Committee on Animal Experimentation (protocol number 14/2020).

Concisely, after a 12-hour fasting period, the animals were anesthetized by a combination of Ketamine and Xylazine Hydrochloride (Francotar^®^ and Virbaxyl 2%^®^, Virbac do Brasil Indústria e Comércio Ltda, Brazil), in the proportion of 0.08 ml/100 g body mass and 0.04 ml/100 g, respectively. After immobilization in ventral decubitus, the animals underwent trichotomy of the inner region of the right and left paws. Antisepsis of the region was performed with sterile gauze soaked in a 10% aqueous solution of povidone-iodine. An incision of approximately 10 mm was performed in planes over the tibial metaphysis, then tissues were dissected. The osteotomy was performed using a 2.0 mm spear drill (Neodent, Curitiba-PR, Brazil), under irrigation with abundant saline solution, with an electric motor adjusted to 1,200 rpm (BLM 600, Driller, São Paulo, Brazil). Subsequently, the implant was installed with a 1.2 mm hexagonal digital key (Neodent, Curitiba-PR, Brazil). Tissue suturing and the postoperative drug protocol used were similar to previously described protocols.^15^

After the periods of 15 and 45 days, the animals were euthanized by anesthetic overdose, and tibias were removed. Left tibias (64 implants) were used for the biomechanical evaluation, via the removal torque test. Then, bone tissue from 15-day samples was used to evaluate gene expression of osteogenic markers via polymerase chain reaction (PCR) array. Right tibias (64 implants) were used in the histomorphometric analysis and three-dimensional imaging (μ-CT) for both periods.

#### Biomechanical test

For left tibias, to maintain the original mechanical strength of the bone tissue, the tibia was removed immediately after euthanasia, then stabilized in a small vice. A compatible wrench was connected to the implants, as well as a torque gauge (Tohnichi ATG24CN-S, Japan). Counterclockwise movements were performed to unscrew the implants and measure the strength of the removal torque (n=16/group).

#### Micro-computed tomography imaging (Micro-CT/μ-CT)

Right tibias were fixed in 4% paraformaldehyde for 48 hours, washed in running water for 24 hours, and stored in 70% alcohol for bone tissue analysis in the μ-CT imaging (Skyscan 1176, Bruker, Aartselaar, Belgium) (n=16/group). The parameter settings were: 65kV, 385μA, 300-ms; isotropic voxel size: 18μm, camera pixel: 12.45). The images were reconstructed, spatially repositioned, and analyzed by specific software (NRecon, Data Viewer, CTAnalyser, Aartselaar, Belgium). The parameters set for reconstruction were artifact correction at 2, beam-hardening correction at 30%, and smoothing at 2%. The region of interest (ROI) was defined as a 0.5 mm circular region around the entire implant diameter, outlined every 10 planes. This ROI was defined as the total volume (0.5 mm margin around implants- 4.5 mm × 3.2 mm). The threshold used in the analysis was 25–90 grayscale and the volume values of the mineralized tissue around the implants were obtained in percentage values. The assessed bone architectural parameters were Bone Volume (BV), Tissue Volume (TV), Bone Volume Fraction (BV/TV), Trabecular Thickness (Tb.Th), Trabecular Separation (Tb.Sp), and Trabecular Number (Tb.N). A trained examiner blinded to the experimental groups performed this analysis.

#### Histological analysis

**Figure 1:**
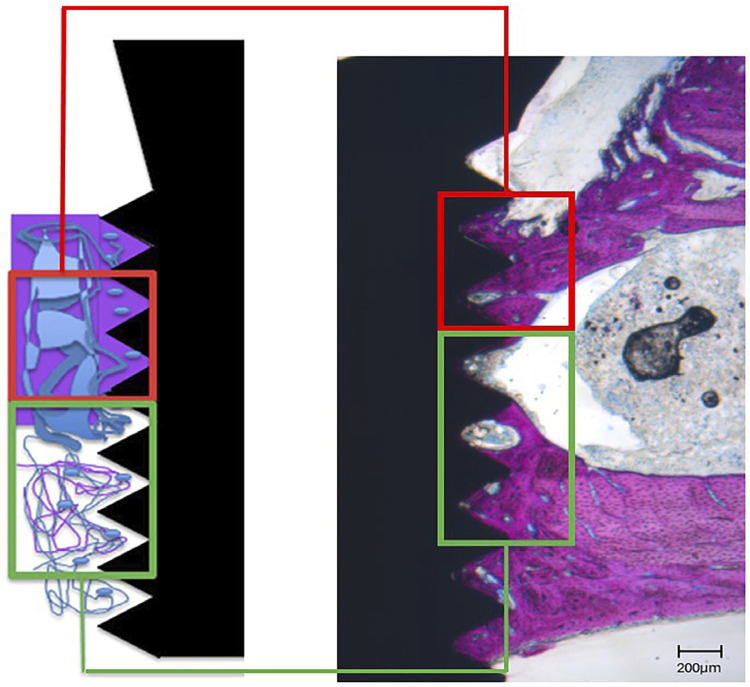
Schematic drawing of the implant with the area represented by the “cortical bone” space measured by two valleys (in red) and the “cancellous bone” space measured by three valleys (in green).

The interface bone tissue response was qualitatively evaluated in the vicinity of implant. After micro-CT scanning procedures, the samples were progressively dehydrated in ethyl alcohol solutions (80%, 90%, and absolute). Plastic infiltration was performed with mixtures of glycolmethacrylate (Technovit 7200 VLC, Kultzer Heraus GmbH & CO, Wehrheim, Germany) and ethyl alcohol following gradual variations, ending with two infiltrations of pure glycolmethacrylate. After plastic infiltration, the slides were embedded in light-curing resin and polymerized. The blocks were mounted on an acrylic slide with the aid of a Tecnovit 4000 resin (Kulzer, Wehrheim, Germany). A cutting–grinding device (Exakt Apparatebeau, Hamburg, Germany) was used to obtain 50 μm slides, which were stained with Stevenel’s Blue. Bone Implant Contact (BIC) and Bone Area Fraction Occupancy (BAFO) were quantified as follows: BIC% = (the extent of bone in direct contact with the implant surface/total length of the implant surface) × 100%; BAFO% = (the area of bone in screw threads of the implant/total area in screw threads of the implant) × 100%. A conventional light microscope (Leica Reichert & Jung products, Germany) at 4/10 ×magnification, coupled with a Leica Microsystems DFC-300-FX camera (Leica Microsystems, Germany) with a resolution of 1.3 megapixels, was used for photographs. The parameters were determined using an image processor (ImageJ 1.45s, NIH, USA). Values for the linear length of bone/implant contact and the bone area were obtained in μm and μm^2^, respectively. The measurements were performed by a trained examiner, calibrated, and blinded to the experimental groups. In total, two distinct regions of threads were considered for the analysis, as shown in Figure 1.

#### PCR Array

The osteogenesis-related gene profile was examined using PCR-Array (RT2 Profiler PCR Array Rat Osteogenesis - catalog no. 330231 PARN-026ZC, Qiagen, USA). After the removal torque test, the remaining bone from the site around the implants of each group was removed, giving a margin of 2 mm from the implant site, stored in liquid nitrogen, and transferred to the −80°C freezer for subsequent RNA extraction. The tissue was macerated in liquid nitrogen, and then total RNA extraction was performed using TRIzol^TM^ Reagent (catalog no. 15596018, Applied Biosystems, Waltham, MA, USA) and PureLinkTM RNA Mini Kit (Applied Biosystems, Waltham, MA, USA), following the manufacturer’s instructions. DNase treatment (RNase-FreeDNase Set - catalog no. 79254, Qiagen, Hilden, Germany) was used to eliminate genomic DNA contamination. The amount and purity of RNA were measured in a NanoVue UV/Visible Spectrophotometer (GE Healthcare, Piscataway, NJ) by assessing the absorbances at 260 nm and the ratio between the absorbances at 260/280 nm, respectively. Then, reverse transcriptase reaction for complementary DNA (cDNA) synthesis was performed using a High-Capacity cDNA Reverse Transcription Kit (catalog no. 4368814, Applied Biosystems, Waltham, MA, USA), following the manufacturer’s protocol. Real-time PCR reaction (n = 5/group) was performed using the StepOnePlusTM Real-Time PCR System (Applied Biosystems, Waltham, MA, USA) at the cycling condition optimized by the manufacturer (10 min at 95°C and 1 min at 60°C). The cycle threshold (CT) values of each well were estimated by the thermal cycler software and exported to a data sheet provided by the Array kit manufacturer. To analyze the data obtained, the CT values were exported to Qiagen’s online website (http://www.qiagen.com/geneglobe). Fold change was estimated by determining the ratio of mRNA levels to control values using the ΔCT method (2^Ct^). All data were normalized to an average of housekeeping genes. A Fold change difference of at least 2, up or down, was considered for selecting the prominent genes (p<0.05).

## In vitro experiments

### Cell culture

For the *in vitro* experiment, the murine pre-osteoblastic cell line MC3T3-E1 (ATCC, USA) was used. Cells were cultured in alpha-minimum essential medium (α-MEM, Invitrogen, Karlsruhe, Germany) supplemented with 10% fetal bovine serum (FBS, Invitrogen), 100 units of penicillin, and 100 μg/ml streptomycin (Invitrogen). The cells were grown at 37 °C in a humidified atmosphere of 5% CO_2_, and the culture medium was changed every other day. MC3T3 cells were seeded (2×10^4^ cells/disc) onto the experimental discs in a 50-μl drop on a 24-well cell culture plate. After 4 h, the final volume of the well was completed to 1 ml. For the experiments, 10 mM β-glycerophosphate (Carl Roth, Karlsruhe, Germany) and 5 μg/ml ascorbic acid (Carl Roth) were added to the medium.

### Real-time PCR

In total, three separate experiments were conducted with a total of nine samples/group/period. RNA extraction was performed using the RNeasy Mini Kit (Qiagen, Hilden, Germany) on the seventh day of cell culture, following the manufacturer’s instructions. To determine the RNA concentration, the NanoDrop ND-2000 spectrophotometer (Thermo Fischer Scientific, Waltham, MA, USA) was used. The iScrip Select cDNA Synthesis Kit (Bio-Rad Laboratories, Munich, Germany) was used to reverse transcribe 500 ng of total RNA, following the manufacturer’s protocol. Gene expression of glyceraldehyde-3-phosphate dehydrogenase (*Gapdh*), bone morphogenetic protein 2 (*Bmp2*), osteopontin (*Spp1*), bone sialoprotein (*Ibsp*), osteocalcin (*Bglap*), runt-related transcription factor 2 (*Runx2*), collagen type I alpha 1 chain (*Col1a1*), and alkaline phosphatase (*Alpl1*) was analyzed by real-time PCR using the PCR thermal cycler CFX96 (Bio-Rad Laboratories), SYBR green PCR master mix (QuantiFast SYBR Green PCR Kit, Qiagen), and specific mouse primers (QuantiTect Primer Assay, Qiagen). Then, 1 µl cDNA was mixed with 12.5 µl master mix, 2.5 µl primer, and 9 µl nuclease-free water. The mix was heated at 95 °C for ٥ min, followed by 40 cycles of denaturation at 95°C for 10 s, and a combined annealing/extension step at 60°C for 30 s. Data were analyzed by the comparative threshold cycle method.

### Protein analyses

Commercially available ELISA kits were used to measure the protein levels of Bmp-2 (Human/Murine/Rat BMP2 ELISA Development Kit, 900-K255, PeproTech, Cranbury, NJ, USA) and Spp-1 (RayBio Mouse OPN ELISA Kit, ELM-OPN, RayBiotech, Peachtree Corners, GA, USA) in cell supernatants according to the manufacturer’s instructions. The optical density was determined using a microplate reader (BioTek Instruments, Winooski, VT, USA) set to 450 nm for Spp-1, whereas for Bmp-2 it was set to 405 nm, with wavelength correction set at 650 nm. For this test, two separate experiments were conducted with a total of six samples/group/period.

## Statistical analysis

Data were expressed as mean ± SD and analyzed in the GraphPad Prism software version 9.0. First, the Shapiro-Wilk normality test and Bartlett’s test were performed. In the presence of data normality and variance homogeneity, parametric tests of mean comparison between groups were performed using analysis of variance (ANOVA)—using one-way ANOVA and two-way ANOVA for the *in vitro* and *in vivo* experiments, respectively—, followed by Tukey’s multiple comparisons test. In the absence of assumptions of normality and homogeneity of variances, the Kruskal-Wallis test followed by Dunn’s multiple comparisons test was performed. The CT values of PCR Array results were directly handled on the company’s website. The significance level was set at α = 0.05, and a P < 0.05 was considered statistically significant.

## Results

### Surface topography characterization

The surface groups investigated during tests were characterized from micro to nano topographic using high-resolution scanning electron microscopy. Microanalysis was performed using x-ray photoelectron spectroscopy to map the strontium content onto the surface of implant materials. Laser scanning confocal microscopy was performed on the surface of the implant specimens aiming to assess the amplitude of surface structures.

**Figure 2:**
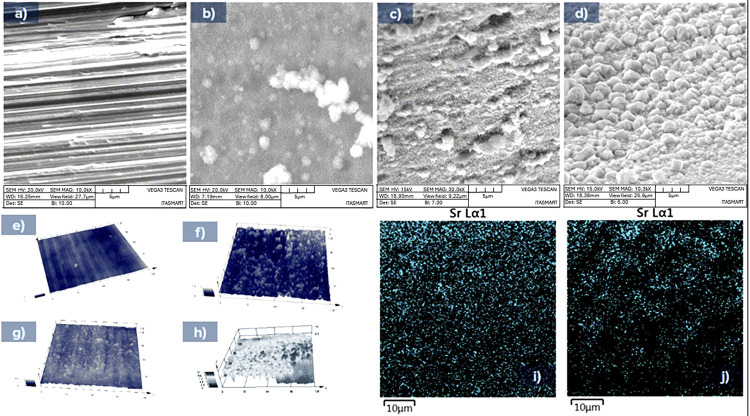
SEM micro images at 10.000 × magnifications of the surface technological groups after treatment on the titanium implants of groups C (a); MN (b), MNSr (c), and MNSr+ (d). Below is Sr mapping by EDS (i; j). e–h show laser scanning confocal microscopy 3D reconstruction images of surface groups. Moreover, e–h shows a representative area of 128 µm × 128 µm, identifying peaks and valleys with at least 200 nm of resolution in height. It is possible to verify the moderately rougher groups MN, MNSr, and MNSr+ compared to the machined conditions.

Control implants showed an isotropic surface configuration with aligned machined marks created during the implant fabrication, as demonstrated in the topography at 10.000× magnification in Figure 2*a* and *e*.

Micro-nano textured surface groups demonstrate an anisotropic configuration of micro to nano-scaled topography, showing micro and nanofeatures well defined in a spread form to guide cells in the regenerative sense, Figure 2*b* and *f*.

Micro-nano topographic surface with discrete additions of strontium (Sr mapping distribution in Figure 2*i*) dispersed along the surface of the implants from this group, Figure 2*c* and *g*.

Micro-nano topography structured surface, Figure 2*d* and *h*, constructed with strontium elements incorporated directly to the surface of implants (Sr mapping in Figure 2j).

### Biomechanical test

**Figure 3:**
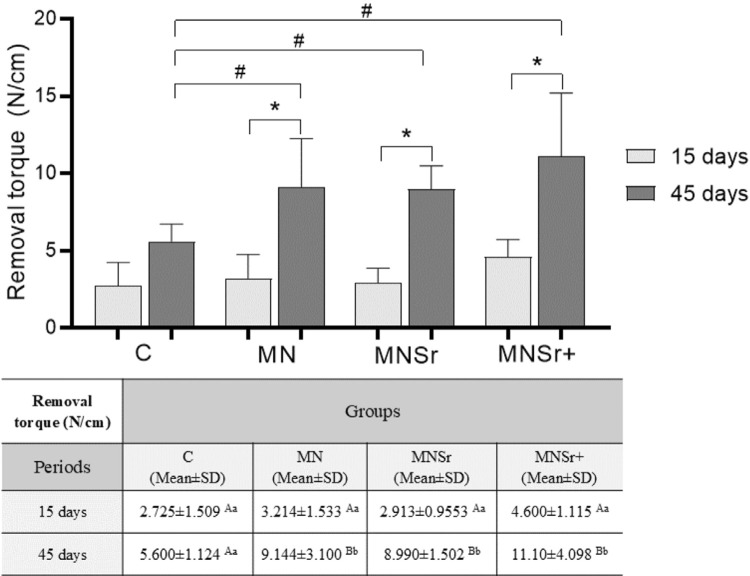
Means and standard deviations (SD) of removal torque analysis in N/cm of implants with different surface treatments at 15 and 45 days. Distinct superscript letters (upper case compares the periods in the column, lower case compares the surface treatments in the row) indicate statistical difference to the Two-Way ANOVA test followed by Tukey (P<0.05). In the graphical representation, * indicates a significant difference between the periods in the same group (P<0.05) and # a significant difference between groups (P<0.05).

Figure 3 and Table 1 display the values and graphic representation of the maximum pull-out forces measured 15 and 45 days following implantation surgery. The treatment groups exhibited significantly higher maximal pull-out forces than the control group at 45 days. Moreover, differences between the two time points were observed within the experimental groups. In contrast, no statistically significant difference was found for the maximal pull-out forces between both periods in the control group.

**Table 1:** Means and standard deviation (SD) of removal torque analysis in N/cm of implants with different surface treatments at 15 and 45 days.

Removal torque (N/cm)	Groups			
Periods	C (Mean±SD)	MN (Mean±SD)	MNSr (Mean±SD)	MNSr+ (Mean±SD)
15 days	2.725±1.509^Aa^	3.214±1.533^Aa^	2.913±0.9553^Aa^	4.600±1.115^Aa^
45 days	5.600±1.124^Aa^	9.144±3.100^Bb^	8.990±1.502^Bb^	11.10±4.098^Bb^

Distinct superscript letters (upper case compares periods in the column, lower case compares surface treatments in the row) indicate statistical difference to the Two-Way ANOVA test followed by Tukey (p<0.05).

### Micro-computed tomography scanning (Micro-CT/ μ-CT)

**Figure 4:**
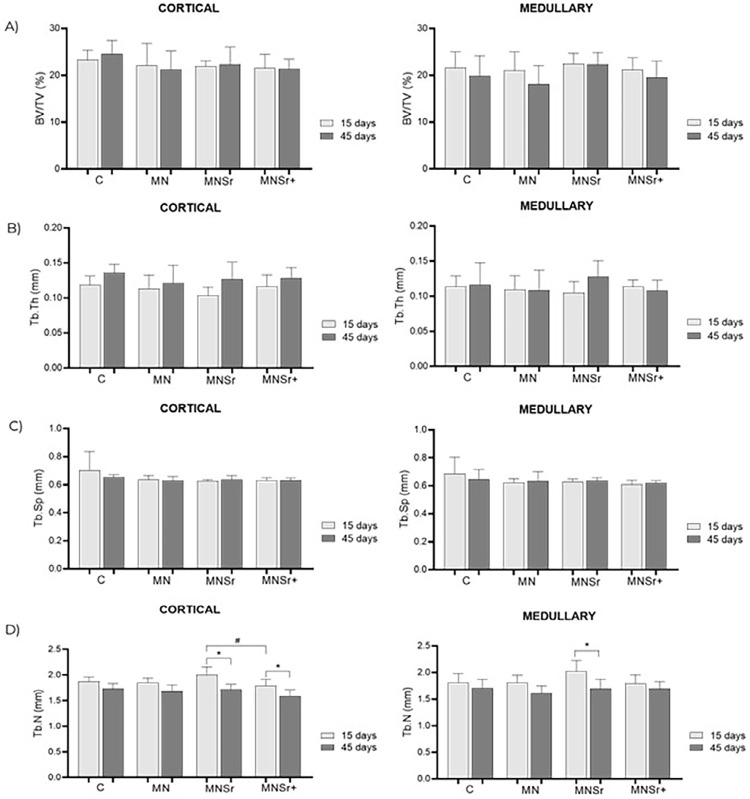
Means and standard deviations of (a) bone volume fraction (BV/TV), (b) Trabecular bone thickness (Tb.Th), (c) Trabecular Separation (Tb.Sp), and (d) Trabecular Number (Tb.N) in the cortical and medullary bone region of implants with different surface treatments at 15 and 45 days.

For the Micro-CT analyses, we discriminated between two evaluation areas, namely the cortical bone area, consisting of the first two threads after the most coronal portion, and the cancellous bone area, corresponding to the central area, in which the first three valleys were excluded, and the subsequent three on each side were included. The results are shown in Figure 4, and the values are shown in Supplementary Tables (Tables 6–13). The results showed no significant differences between the treatments concerning BV/TV, Tb.Th, and Tb.Sp. However, a statistically significant decrease in Tb.N was observed between the periods in the Sr-doped groups for the cortical bone area and in the MNSr group for the medullary bone area (Figure 4d).

### Histological evaluation and bone-implant contact percentages

**Figure 5:**
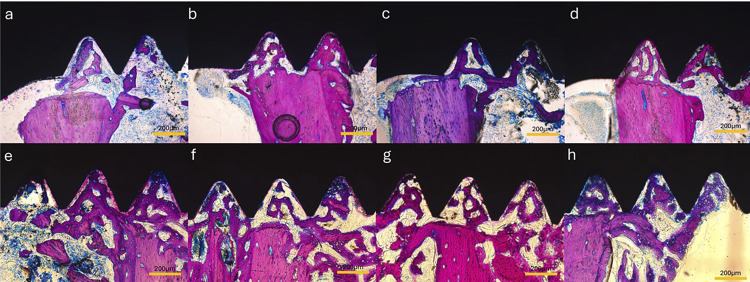
Histological slides for all examined surfaces. (a–d) cortical thread region and (f–g) medullary thread region. (a, e) Control; (b, f) MN surface; (c, g) MNSr surface; (d, h) MNSr+ at 15 days. Magnification at 10×.

At 15 days post-implantation, all implants in both the control and experimental groups exhibited histological evidence of direct bonding with the adjacent bone, without indications of inflammation at the interface between bone and implant (Figure 5).

**Figure 6:**
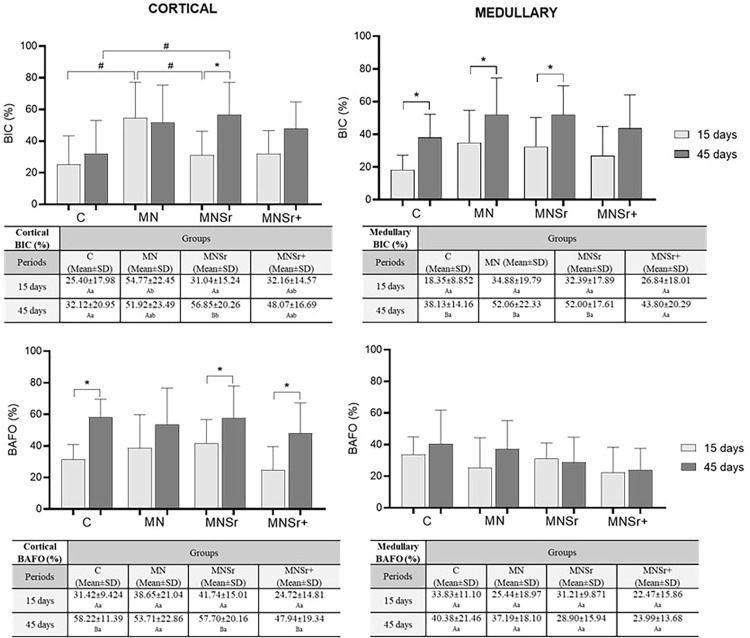
Histometric analysis comprising BIC and BAFO measurements in the regions corresponding to the cortical and medullary bones. Distinct superscript letters (upper case compares the periods in the column, lower case compares the surface treatments in the row) indicate statistical difference by the two-way ANOVA followed by the Tukey’s test (p<0.05). In the graphical representation, * indicates a significant difference between the periods in the same group (p<0.05) and # a significant difference between groups (p<0.05).

Figure 6 shows the BIC and BAFO results. The BIC increased in cortical bone area in the treated groups compared to the control, although a significant difference (p<0.05) was observed only for the MN and MNSr groups. A significant increase in BAFO was also observed in the cortical bone area between the periods for the C, MNSr, and MNSr+ groups.

### PCR Array

**Figure 7:**
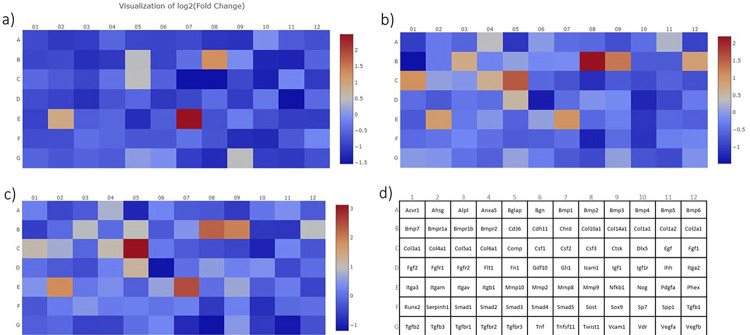
Heat Maps demonstrating the comparison and fold changes of the genes diagram of genes associated with osteogenesis ofgroups. Fold change comparisons between MN (a), MNSr (b), and MNSr+ (c) groups in contrast to the control group. The layout of thePCR array can be observed in (d)

**Figure 8:**
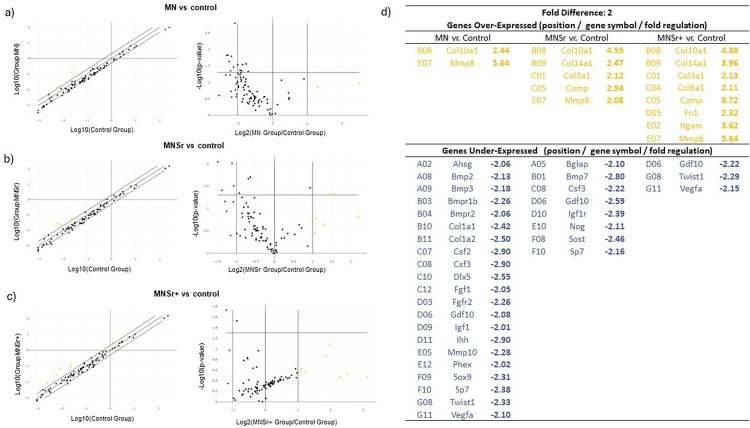
Scatter Plot generated using Qiagen’s PCR Array Data Analysis Web Portal, which serves to contrast the normalized expression levels of individual genes on the PCR Array between the treated experimental groups in relation to the control group. In this representation, genes exhibiting upregulation are situated in the upper left quadrant denoted by yellow dots, whereas genes displaying downregulation are located in the lower right quadrant represented by blue dots. The black dots represent genes whose expression remains unchanged. Moreover, the accompanying Volcano Plots on the right-hand side depict statistical significance, measured in terms of P-values, juxtaposed with the extent of change, expressed as fold change, observed in the analyzed genes. These comparisons specifically pertain to the fold change values between MN (a), MNSr (b), and MNSr+ (c) groups in contrast to the control group. Detailed values of fold changes, accompanied by their respective statistical significance, are presented in panel (d).

The quantification of baseline osteogenic markers is valuable in confirming the osteogenic differentiation potential of bone cells on new titanium surface treatments, although it is insufficient to reveal the underlying complexity of the osteogenic pathway. Among the focused panel of 84 genes related to osteogenic differentiation (Supplementary Table 14), the surfaces affected gene expression differently (Figure 7). The micro-nano textured surface resulted in the upregulation of two genes, whereas 21 genes were downregulated (Figure 8) on day 15. The Sr-doped surface, on the other hand, regulated a smaller number of genes than the micro-nano textured surface. We highlight that, when comparing the Sr-doped groups, the MNSr group upregulated fewer genes and downregulated more genes than the MNSr+. Meanwhile, the MNSr+ group showed the opposite behavior. These regulated genes belong to functioning gene groups that cover almost all aspects of osteogenesis development.

### MC3T3-E1 culture

**Figure 9:**
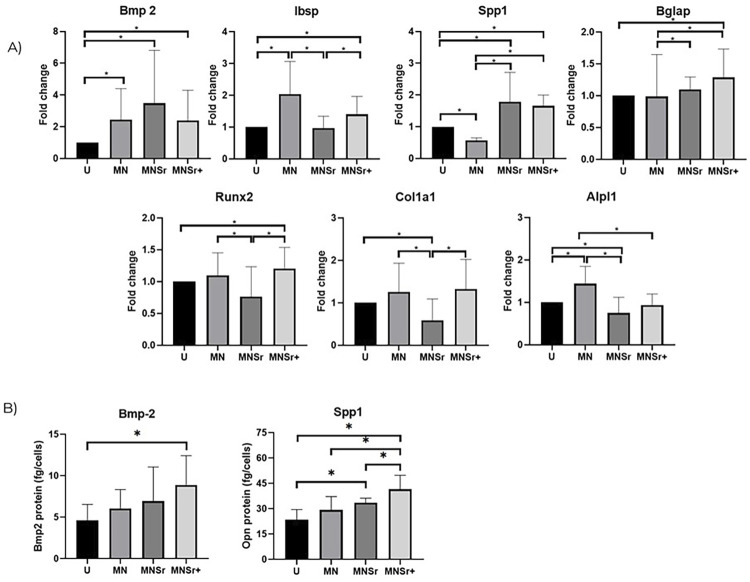
A. Fold change graphs of genes related to the osteogenic pathway evaluated at the 7-day timepoint. B. ELISA test of culture medium collected from cell supernatants after 7 days of cell culture. The concentration was expressed in pg/ml and normalized according to the number of cells (fg/cells). * indicates statistical difference between groups by the Kruskal-Wallis test followed by the Dunn’s multiple comparisons test (p<0.05).

Fold change differences in RNA expression of proteins associated with osteogenesis were observed between treatments for all molecules analyzed at 7 days of cell culture, as shown in Figure 9a. In brief, MCT3-E1 cells culture on tread surfaces exhibited regulated expression with increasing trends of *Bmp-2, Ibsp, Spp1, Bglap, Runx2, Col1a*, and *Alp1*. In the protein evaluation at 7 days of culture, ELISA results were normalized according to the number of cells and showed higher expression (p<0.05) of Bmp-2 in the MNSr^2^ group and Spp-1 in both Sr-doped groups compared to the control (Figure 9b), as well as increased gene expression of Bmp-2 and Spp-1. These results suggest that surface treatments, especially those doped with released Sr^2^ions, facilitate early osteogenesis of MCT3-E1 cells *in vitro*.

## Discussion

This study demonstrated, both *in vivo* and *in vitro*, the beneficial biological effects of implant surface texturing at the micro- and nano-scales, as well as functionalization with strontium. This metallic element in ionic form has been shown to regulate bone metabolism as a dual-action agent, stimulating both pre-osteoblast and osteoblast differentiation, as well as inhibiting osteoclast differentiation and activation.^9,16,17^ This regulation of bone remodeling is widely recognized as effective, even in the context of peri-implant regeneration.^9^ Recent systematic reviews have shown that local administration of strontium from functionalized implant surfaces allows for higher localized dosing, avoiding systemic side effects, and improving implant osseointegration.^12^

In general, this study demonstrated that implants with treated surfaces exhibit osteogenic properties due to the micro- and nano-scale structures and the biological effects of Sr, observed by the biomechanical, histomorphometric, and gene expression results, which are consistent with numerous other evaluations.^6,8,18^ In our study model, removal torque values demonstrated superior effects on bone formation when compared to machined implants, implying enhanced early-stage osseointegration of the treated implants. A systematic review and meta-analysis^14^ examined the impact of strontium incorporation into titanium implants on bone apposition when implanted in animal models. In total, nine of the included studies reported superior biomechanical test results on strontium-incorporated surfaces compared to control ones, with substantially higher removal torque values, maximum push-out force, and/or shear strength.

In this study, histomorphometric analysis revealed superior results for BIC% in the cortical threads of the functionalized surfaces at 15 and 45 days. This finding is of great significance, as BIC% is widely recognized as the gold standard for assessing bone formation around metallic implants. Contact osteogenesis has been shown to play a pivotal role in the success of implant therapy.^3^ These results may be interpreted as accelerating the bone healing process, which can be particularly beneficial in complex clinical scenarios.^1^ Experimental implants containing Sr^2^ produced by hydrothermal treatment also showed significantly higher BIC percentages in rabbit tibias and femurs compared to control implants.^7,18,20^On the other hand, the BAFO results in our study yielded comparable results among the groups after both time intervals. These findings support the hypothesis of a localized effect measurable near the surface due to the direct release of strontium at the implant-tissue interface, as no significant differences were observed at a considerable distance from the surface in the period investigated. Similarly, other studies have found analogous findings assessing late-stage osseointegration of Sr-functionalized surfaces in healthy animal models at 6^7,21^ and 12 weeks.^6^ In these reports, no statistical differences were observed regarding bone area, whereas an increase in BIC was evidenced for the functionalized surfaces compared to controls. Our findings revealed a significant increase in the BIC% value just two weeks after implantation, underscoring the crucial role of micro- and nanotextured surface treatments in accelerating the osseointegration of implants.

Shi, et al.^14^ (2017), in their review, also examined studies that employed μ-CT in quantitative analyses. Among the included studies, four revealed that strontium-enhanced implants exhibited a more substantial impact on all μ-CT parameters when compared to control implants. Meanwhile, three other studies did not find statistically significant differences in some of these parameters.^14^ Our study also observed comparable group results between the two time periods for most of the analyzed parameters. However, a statistically significant decrease in Tb.N in the cortical and cancellous bone area of the MNSr group and in the cortical area of the MNSr+ group between the two evaluation periods was observed. This can be justified by increased bone formation and trabecular fusion, rendering the bone denser and with a reduced number of trabeculae detected by μ-CT.

The μ-CT results generally exhibited similar trends to the histomorphometric analysis, although statistical significance was not reached. It is worth noting, however, that information loss during sectioning and histological preparation procedures can lead to uncertain outcomes, as previously described.^17,22,23^ Additionally, μ-CT analysis shows disadvantages, such as metallic artifacts caused by a combination of beam hardening, scatter, and non-linear partial volume effect. Thus, while it is a desirable method for measuring bone parameters, special caution is required when assessing osseointegration due to various limitations.^22,23^ Differences between μ-CT and histology may be observed mainly due to metal-induced artifacts on μ-CT systems or partly because of substantial intra-group variations (up to 35%) among histological sections^24^, which was not observed in the present study. Moreover, the concurrent evaluation of removal torque is opportune to assess a potential correlation between increased bone apposition in the implant vicinity and potential improvement in mechanical stability. Regarding the promotion of the osseous response by functionalized surfaces, for our treated and doped-strontium surfaces, the results of BIC% and removal torque, when considered together, suggest an acceleration of osseointegration.

Another distinctive feature of our study was the in-depth assessment of osteogenesis-related bone markers using the PCR Array technique. In total, 84 markers associated with this pathway were evaluated for the 15-day period to elucidate the transcriptional events in the early phases of osseointegration. We highlight the upregulation of certain genes in the Sr-doped groups. A total of eight genes were upregulated in the MNSr+ group, whereas five genes exhibited upregulation in the MNSr group. Although greater amounts of Sr promoted the upregulation of more osteogenic genes at 15 days, Sr in greater quantities at the surgical site was not reflected in clinical osseointegration results. Except for a slight increase in TbN at 15 days in the Mn group compared to the MnSr++ group, no significant differences were observed between the MnSr and MnsSr groups in the removal torque and the histological and histomorphometric analyses. Sila-Asna^25^ (2007), when conducting *in vitro* experiments, found optimized concentrations of strontium ranelate in hMSCs and osteoblastic cells ranging from 0.2107 to 21.07 ppm. However, a tenfold higher concentration (210.7 ppm) caused a negative effect, decreasing the expression of bone sialoprotein and a delayed effect on osteoblastic differentiation with delayed expression on Cbfa1 and osteonectin.^24^

Among the upregulated genes, matrix metalloproteinase 8 (*Mmp-8*) stands out, being overexpressed in all three treatment groups. Also known as collagenase-2, it is an enzyme that plays a role in extracellular matrix remodeling and the body’s inflammatory response. MMP-8 substrates include collagen I, II, III, and IV, among the important proteins in the soft tissue surrounding implants.^26^ Moreover, MMPs can modulate bone resorption via the activation and differentiation of osteoclasts, in addition to the direct degradation of bone collagen matrix. Thus, even though MMP-8 is not typically directly associated with bone formation, it may play an important role in regulating the extracellular matrix, as it is involved in the degradation and remodeling of collagen during physiological processes such as wound healing and bone remodeling.^26^ Interestingly, collagen markers were also upregulated in the treated groups, such as Collagen type X alpha 1 (*Col10a1*), Collagen type XIV alpha 1 (*Col14a1*), Collagen type III alpha 1 (*Col3a1*), and Collagen type VI alpha 1 (*Col6a1*), suggesting increased bone remodeling activity in the treated groups, especially in the MNSr+ group.

Integrin subunit alpha M (*Itgam*), a gene related to cell adhesion molecules and cell-matrix adhesion,^29^ was upregulated in the MNSr+ group (3.62-fold). Fibronectin 1 (*Fn1*) also showed increased expression in this group (2.32-fold). This gene plays a significant role in various biological processes, including cell migration, cell adhesion, and cytoskeletal organization. Previous reports have indicated that osteoblasts produce FN-1 during the stages of proliferation and differentiation, concomitant with the synthesis of collagen type I, suggesting that osteoblasts generate FN-1 during active bone formation processes.^30,31^

Many downregulated genes were observed in the treatment groups compared to the polished control, and these genes are associated with various functions, including cell adhesion and growth differential, skeletal development, bone mineralization, ossification, and the production of extracellular matrix proteins. The 15-day time point can be relatively late when attempting to assess differences in gene expression during the initial phase of cell differentiation and bone formation around the implant. Therefore, the negative regulation of osteogenic markers in the treated groups can indicate that the biological osteogenesis-related events were anticipated since many osseointegration events had already occurred in the first-week post-implantation at the surgical site. Thus, the 15-day period may be untimely to capture the peak of positive regulation of early adhesion and osteogenesis markers, which may have been slower on the machined surface.

Therefore, investigations conducted at shorter intervals could show a greater capacity to highlight distinctions between the surfaces subjected to treatment. In this context, a supplementary study was conducted *in vitro* using discs that underwent the same surface treatments for seven days regarding gene expression and protein expression of osteogenic markers. BMP-2 is one of the key cytokines involved in regulating the differentiation and maturation of precursor cells into osteoblasts,^32^ and its expression was increased in all treated surface groups (p<0.05) (Figure 9a). Similarly, an increase in the protein expression of this marker was also observed via ELISA assays on micro-nano textured surfaces. However, a statistically significant increase was only observed for the MNSr+ group (Figure 9b). Interestingly, Bmp-2 was upregulated at 15 days in the *in vivo* study for the MN group, corroborating our hypothesis that downregulated genes observed in the PCR surpassed the pronounced expression due to the advancement of effects induced by the surface treatments in implant. Also, favorable regulations of Bmp-2 on Sr-loaded surfaces were observed in other studies.^33,34^ Similar to *Bmp-2, Spp1*, which is involved in the synthesis and regulation of osteopontin, showed increased expression in the Sr groups, as well as protein quantification (Figure 9b). This protein is essential in regulating the extracellular matrix, bone mineralization, the immune response, and various biological processes comprising osseointegration.^35^ Geng, et al. (2022)^34^ also obtained similar results and observed that Sr-loaded surfaces promoted increased osteopontin expression *in vitro* at four and seven days.

Osteocalcin, also known as bone gamma-carboxyglutamic acid-containing protein (*BGLAP*), is the most abundant non-collagenous protein found in bone and is commonly used as a biomarker of bone turnover.^35,37^ This gene also showed increased expression in the Sr-doped groups, especially in the MNSr+ group, which showed superior expression compared to both the control (p=0.0317) and the standard micro-nano textured substrate (p<0.0034), highlighting the positive aspects of Sr^2^ ion release in modulating non-collagenous proteins. Similarly, collagen type I, the primary protein of the organic matrix (80–90%)^37^, despite no statistical significance, presented a tendency for expression in the micro-nano surface group at 7 days. In the *in vivo* results at 15 days, it was already upregulated compared to the control (−2.42 FC; Figure 8*d*). In the MNSr group, the expression was reduced compared to the other groups, and at 15 days in the *in vivo* study, it was similar among the groups.

The combined micro- and nanoscale modification of titanium implants demonstrates beneficial conditions for osteogenic cell growth since a random dimensional structure mimics the hierarchical structure of bone tissue.^39^ Our findings run along these lines. Although the removal torque and histomorphometric evaluations show the benefits of surface treatments at the nanoscale level, the results of the *in vivo* and *in vitro* gene analyses also suggest chemical benefits, with strontium enhancing the effect of micro-nano topography.

The potential for improved bone formation by incorporating Sr into biomaterials has also been well-documented in both *in vitro* and *in vivo* studies.^6^ However, we highlight some limitations in this study. For example, *in vitro* assays using only one cell type, as employed in our study, hold potential to assess individual biological processes in a controlled manner but cannot fully replicate the complex *in vivo* environments, especially when investigating the effects of multipotent drugs such as Sr^2^ Thus, animal models are complex studies with multiple variants and provide important highlights found *in vitro*, despite the limitations imposed by the implantation site (long bone instead of maxillary) and euthanasia periods, intrinsic for surgery protocols. Another limitation in this study relates to not assessing how long the release of Sr in the peri-implant tissue lasted. However, a previous report has attested to the sustained Sr release from a doped surface by hydrothermal treatment for more than 14 days.^40^ Likewise, it is interesting that further studies also focus on the long-term stability of these implants to determine precisely how long these coatings last and establish whether they are stable and remain so between the production process and their final destination.

## Conclusion

Our findings, taken together, suggest that micro-nano surface texturization combined with strontium loading can synergistically improve both bone microarchitecture and the biological response of the host bone, thus achieving enhanced osseointegration at the nano level.
